# Correlation of diaphragmatic ultrasound with pulmonary function testing in patients with chronic cervical spinal cord injury: A single center pilot study

**DOI:** 10.1080/10790268.2025.2534262

**Published:** 2025-07-31

**Authors:** Shane N. Stone, Sungchul Huh, Jayme O’Connor, Lauren Murphy, Steven Kirshblum

**Affiliations:** 1 Department of Physical Medicine and Rehabilitation, UC Davis Health, Sacramento, California, USA; 2 Department of Physical Medicine and Rehabilitation, Pusan National University Yangsan Hospital, Yangsan-si, Gyeongsangnam-do, South Korea; 3 Department of Multiple Myeloma, Memorial Sloan Kettering, New York, New York, USA; 4 Kessler Institute for Rehabilitation, West Orange, New Jersey, USA; 5 Department of Physical Medicine and Rehabilitation, Rutgers New Jersey Medical School, Newark, New Jersey, USA; 6 Kessler Foundation, West Orange, New Jersey, USA

**Keywords:** Spinal cord injuries, Diaphragm, Diagnostic imaging, Ultrasonography, Pulmonary function tests

## Abstract

**Context/Objective:**

Pulmonary function tests are the gold standard of evaluating pulmonary function in individuals with a spinal cord injury (SCI). However, there are limitations to its accessibility thereby reducing its clinical utility. Diaphragmatic ultrasound is used to evaluate pulmonary function in non-neurologic individuals but there is limited evidence demonstrating its benefit in individuals with SCI. The objective is to determine if diaphragmatic ultrasound measurements are correlated with forced vital capacity (FVC), peak cough flow (PCF), neurological level of injury (NLI), and/or motor level.

**Design & Setting:**

A pilot study at a single rehabilitation hospital.

**Participants:**

Individuals ≥18yo with traumatic and non-traumatic SCI for ≥6 months with NLI from C1-C6, and ASIA Impairment Scale scores A, B, or C.

**Interventions:**

N/A.

**Outcome Measures:**

Associations between FVC, PCF, and diaphragmatic ultrasound measurements (thickness at total lung capacity, thickness at functional residual capacity, and right diaphragm excursion). Also, associations between diaphragmatic ultrasound measurements with NLI and motor level.

**Results:**

Significant associations were detected between the thickening ratio and FVC (*r* = 0.5384, P = 0.047), diaphragm excursion with FVC (*r* = 0.5757, P = 0.0395), and diaphragm excursion with PCF (*r* = 0.8061, P = 0.009). Thickness at functional residual capacity and thickness at total lung capacity were not significantly associated with FVC nor PCF. No significant association was found between NLI or motor level with any of the recorded pulmonary or diaphragmatic measures.

**Conclusion:**

Diaphragmatic ultrasound is a promising tool in the evaluation of two parameters of pulmonary function in those with cervical SCI. A larger, longitudinal study to confirm these findings and evaluate for normative values would be beneficial.

## Introduction

Respiratory complications are the leading cause of death in individuals with chronic spinal cord injury (SCI) (1), highlighting the necessity to find ways to monitor pulmonary function in the aging SCI population (2). With the rise in accessibility to ultrasound (especially in Physical Medicine and Rehabilitation [PM&R] clinics), the demonstrated cost effectiveness of the modality (3), and research supporting its utility in acute care pulmonary evaluation (4), it is imperative to determine if this tool has potential use for surveillance of pulmonary function in chronic SCI. Furthermore, it would be beneficial to establish normative data for individuals with various levels of SCI, as this will be key to its incorporation into the spectrum of care.

Pulmonary function tests (PFTs) are the gold standard to evaluate an individual’s (with and without SCI) pulmonary abilities and can help to determine readiness to wean off mechanical ventilation (5–8). While there are no definitive guidelines for SCI pulmonary surveillance, the American College of Chest Physicians recommends individuals with neurologic disease at risk for respiratory failure should have PFTs every 6 months (9) though a single longitudinal study assessed annually (10). Unfortunately, with the challenges of accessing subspecialty care (11) and the extended duration of PFTs, this is not a feasible option for most individuals who would most benefit from this surveillance. Fortunately, previous research of PFTs has demonstrated that forced vital capacity (FVC) is correlated with several other PFTs including inspiratory capacity, total lung capacity, functional residual capacity, expiratory reserve volume, and residual volume (12). Additionally, peak cough flow (PCF) (a global measure of cough strength (13,14)) has demonstrated utility in evaluating successful extubation and decannulation in those with neuromuscular disease (8). Therefore, these two PFTs have become the commonly monitored values to evaluate pulmonary health in this population. In SCI, the use of the “Sniff test” (videofluoroscopic evaluation) as well as electrodiagnostic studies have also been used to evaluate pulmonary capacity by determining the strength and degree of innervation of the diaphragm (6,15–17) when there is concern for paralysis, but are less commonly used because of potential complications.

More recently, physicians have started to use and evaluate the utility of diaphragmatic ultrasound as a tool to evaluate an individual’s candidacy for ventilator weaning in those with (18) and without SCI (4) because it is a simple, inexpensive, accessible way to assess pulmonary function that may also act as a more direct evaluation of diaphragmatic function than PFTs or videofluoroscopy (19). If the measurements collected during diaphragmatic ultrasound are correlated with FVC or PCF, then it would provide another tool that can be used to evaluate pulmonary ability in patients with SCI and used for surveillance in an aging SCI population. Zhu *et al.* (20) explored this, but looked at acute injury and did not assess PCF. Additionally, the current normative values used during these ultrasound evaluations are based on healthy individuals (19,21). Though early research has used these normative values, it would be beneficial to determine these values in the SCI population since a few studies have found individuals with SCI tend to have a thicker diaphragm with a greater degree of diaphragm excursion (20,22,23). Since diaphragmatic function is impacted by neurologic integrity, there may be different normative values depending on the neurological level of injury (NLI) or motor level.

The objective of this study was to determine if ultrasound measurements of the diaphragm are correlated with FVC or PCF, NLI, and/or motor level that would warrant further investigation. We hypothesized that there would be a correlation between (1) FVC and diaphragm thickening ratio, (2) PCF and diaphragm excursion, and (3) motor level and thickening ratio.

## Study design and methods

### Setting, participants, and recruitment

Data collection for this study took place from March to May 2024 at a single, SCI rehabilitation hospital. The Institutional Review Board approved this pilot study.

Individuals 18 years of age or older with chronic SCI (≥6 months after injury), with NLI C1-C6, and American Spinal Injury Association Impairment Scale (AIS) scores A, B, or C were included. Exclusion criteria included individuals who were not fluent in English, were cognitively impaired, had other pulmonary comorbidities (including phrenic neuropathy, chronic obstructive pulmonary disease, uncontrolled asthma, interstitial lung disease), had congestive heart failure, were ≥ 4 months pregnant (due to the impact of pregnancy on diaphragm function (24)), required mechanical ventilation, and/or had a phrenic or diaphragmatic pacing system. Of note, participants with current or previous smoking history were not excluded because previous research found no difference in diaphragm measurements between current, previous, and nonsmokers (19).

Eligible participants were identified via chart review and then screened for eligibility via telephone with a trained research coordinator prior to initiating enrollment procedures. If interested, the caller confirmed inclusionary criteria and consented individuals for participation.

### Outcome measures

Demographic characteristics measured included current age, sex at birth, sex, smoking history, NLI, motor level and number of years injured. Motor level was collected in addition to NLI because previous literature has found that motor level (compared to NLI) better reflects the degree of function as well as the severity of impairment after motor complete tetraplegia (25).

All PFTs were collected with participants sitting upright in their wheelchairs. Abdominal binders were removed, as needed, to measure unassisted pulmonary function. Consistent with prior research, each PFT (FVC and PCF) was recorded three consecutive times, with opportunities for rest between each, and then the maximum value was used for analysis (8,26,27). PCF (L/min) was measured using the Strive® dual zone peak flow meter and FVC (mL) using the Aspire Haloscale® Standard. PFTs were collected by the lead author, a PM&R physician with over three years of experience with diaphragmatic ultrasound.

The same physician performed all the scans. Each participant had their diaphragm evaluated in a seated position at a 45° recline to optimize diaphragm function (28,29) and to be consistent with previous research (18). As previous studies have demonstrated the impact of position on measurements, it was decided to record all measurements in the same position (29,30). This was performed in their wheelchair or on an examination table to ensure appropriate positioning and participant comfort. As previously described in Patel and colleagues (31), the thickness of the hemidiaphragm at maximal inspiration (*i.e.* total lung capacity, thickness at total lung capacity) and at end expiration (*i.e.* functional residual capacity, thickness at functional residual capacity) were measured at the zone of apposition (8th or 9th intercostal space) in the bilateral midaxillary views using B-mode. Using these measurements, the thickening ratio was calculated, where thickening ratio = thickness at total lung capacity / thickness at functional residual capacity. The diaphragmatic excursion was measured from the right, midclavicular, subcostal window using M-mode. A previous study noted the technical difficulty of left sided diaphragm excursion in healthy individuals, so it was not attempted in this study (32).

All scans were performed on a single, Sonosite X-Porte Ultrasound. A HFL50xp-15-6MHz-rectangular probe was used for the axillary views and a C60xp 5-2 MHz – fan-shaped probe was used for the subcostal view.

Participants received a $50 gift card at the completion of the study.

### Analysis

Consistent with the American Thoracic Society (ATS) Guidelines for reproducibility FVCs values >150cc and PCF values >20 L/min in the same participant were treated as outliers and the maximum valid values were retained for analysis (33,34). Similarly, symmetry of diaphragmatic ultrasound measurements was assessed using Boon and colleagues (19) that suggests the mean difference of side-to-side thickening ratio had a 95th percentile value of 1.3. Therefore, values beyond this were considered asymmetric.

All data analysis was conducted with Stata/SE version 18. Descriptive statistics were used to summarize all measures. For the correlations, averages of the side-to-side thickening ratios were used by averaging the left and right sided measurements and making a ratio of those averages (35). Normality was assessed for all measures using Shapiro–Wilk tests and kernel density plots to check the appropriateness of using Pearson correlation. Then Pearson correlation coefficients were performed to evaluate for associations between the PFTs and diaphragmatic ultrasound measurements. Spearman rank correlation was performed to evaluate for an association between NLI and motor level with PFTs and/or diaphragmatic ultrasound measurements. Individuals with asymmetric motor levels had the lower level used for comparison (*e.g.* if left was C4 and right C6, C6 was used). This is based off previous literature which demonstrated in individuals with asymmetric diaphragm function that the “best side” was more functionally relevant (35). Significance was assessed using P < 0.05. Coefficients <0.5 were considered “weak,” 0.5–0.6 “moderate,” and >0.8 “strong.”

## Results

### Demographics

Fourteen individuals were enrolled in the study (Table 1). Twelve individuals had traumatic injuries and two had neurologically stable, non-traumatic injuries. The participants with non-traumatic injuries had transverse myelitis and a history of epidural abscess and both presented similarly to those with traumatic injuries. Nine participants were male and five females, all cis sex. The average age was 40.9 years (SD 16.2) ranging from 19–74 years old. NLI of C4 was most represented (7/14), with nearly all participants having a motor level between C4-C6 (10/14), and 13/14 had motor complete injuries (AIS A and B). All patients had suspected sparing of the diaphragm including the one patient with NLI of C2 because their motor level was C6. None of the patients had tracheostomies. Smoking history was variable among participants though 10/14 had a history of tobacco/marijuana use (current or previous use).

**Table 1:** Participant characteristics including age, sex, time since injury, NLI, motor level, AIS grade, etiology of injury, and smoking history.

Participant characteristics	Mean (SD) or n (%)	Min	Max
Age (years.)	40.9 (16.2)	19	74
Sex (%)			
Female	5 (35.7)		
Male	9 (64.3)		
Time since injury (years.)	9.9 (9.9)	1	38
Neurological level of injury (NLI) (%)			
C2	1 (7.1)		
C4	7 (50.0)		
C5	3 (21.4)		
C6	3 (21.4)		
Motor level (%)			
C4	4 (28.6)		
C5	5 (35.7)		
C6	4 (28.6)		
C7	1 (7.1)		
ASIA Impairment Score (AIS) grade (%)			
A	7 (50)		
B	6 (42.9)		
C	1 (7.1)		
Injury etiology (%)			
Traumatic	12 (85.7)		
Non-traumatic	2 (14.3)		
Smoking history (%)			
Currently	3 (21.4)		
Previously	7 (50)		
Never	4 (28.6)		

### PFTs and diaphragmatic ultrasound measurements

PFT assessments were completed for 100% of participants. Using ATS reproducibility guidelines (33,34), one vital capacity and three PCFs were removed and three PCFs (Table 2).

**Table 2:** Summary of all pulmonary function tests and diaphragm ultrasound measurements included in the analysis.

Pulmonary and Diaphragm Outcomes	Mean (SD) or n (%)	Min	Max
*Pulmonary function tests*			
Forced vital capacity (mL)	2250 (818.2)	840	3770
Peak cough flow (L/min)	244.3 (81.8)	130	410
*Diaphragmatic ultrasound measurements*			
Thickness at functional residual capacity (cm)	0.2 (0.1)	0.1	0.3
Thickness at total lung capacity (cm)	0.4 (0.2)	0.2	0.7
Thickening ratio	2.2 (0.4)	1.6	3
Diaphragmatic excursions (cm)*	2.1 (1.3)	0.6	4.5

*Sample size for excursions is 13.

Ultrasound evaluations were completed for all participants though diaphragm excursion was only performed on 13/14. Three individuals had significant right to left side thickening ratio asymmetry (values of 1.37, 1.45, 1.35), however, their data was included given the small sample size and because individuals with tetraplegia often have asymmetry in their injuries (36) which is different than the source of normative values (19).

### Correlations

**Figure 1:**
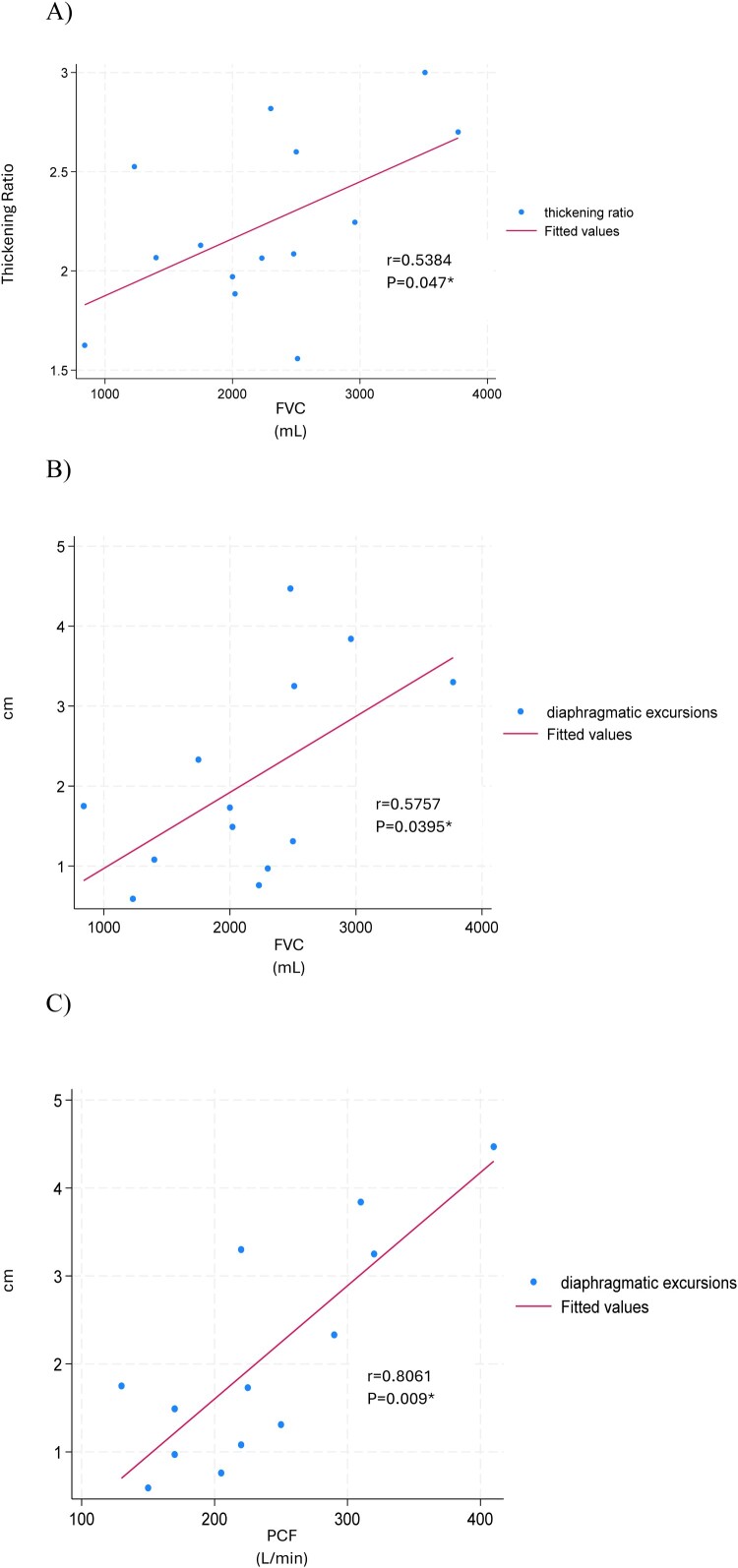
Scatterplots of the significant correlations. (A) Between thickening ratio and FVC, (B) between diaphragm excursion and FVC, and (C) between diaphragm excursion and PCF. *Denotes significance (p < 0.05). FVC: forced vital capacity; PCF: peak cough flow.

Table 3 presents the correlation coefficients for diaphragm average measurements (thickness at functional residual capacity, thickness at total lung capacity, thickening ratio, and diaphragm excursion) and each PFT (Table 3). There was no significant association between thickness at functional residual capacity and thickness at total lung capacity and the PFTs. A significant, moderate correlation was observed between the thickening ratio and FVC (*r* = 0.5384, P = 0.047; (Fig. 1(a))). Diaphragm excursion was moderately correlated with FVC (*r* = 0.5757, P = 0.0395; (Fig. 1(b))) and strongly correlated with PCF (*r* = 0.8061, P = 0.009; (Fig. 1(c))).

**Table 3:** Pearson correlations between the diaphragmatic ultrasound measurements and pulmonary function tests.

Diaphragm Ultrasound Measurements		Pulmonary function tests	
		*FVC*	*PCF*
Thickness at functional residual capacity	Correlation coefficient (r)	0.2008	0.1439
	p- value	0.4913	0.6237
Thickness at total lung capacity	Correlation coefficient (r)	0.3678	0.0731
	p- value	0.1957	0.8037
Thickening ratio**	Correlation coefficient (r)	0.5384	0.07
	p- value	0.047*	0.8121
Excursions***	Correlation coefficient (r)	0.5757	0.8061
	p- value	0.0395*	0.0009*

Note: *Denotes significance (p < 0.05). **Thickening ratio used for correlation was the averaged thicknesses at total lung capacity and thickness at functional residual capacity. ***Sample size for excursions is 13. FVC, forced vital capacity; PCF, peak cough flow.

There was no significant association between NLI nor motor level with any of the recorded PFTs or diaphragmatic ultrasound measurements (Supplemental Material e-Table 1).

Finally, we replicated the analysis after removing participants with non-traumatic injuries as a potentially influential subgroup. Welch’s t-tests were used to compare the mean values of all PFTs and diaphragmatic ultrasound measurements in participants with traumatic (*n* = 12) and non-traumatic injuries (*n* = 2). We find that the differences between the means of the PFTs and diaphragmatic ultrasound measurements for traumatic and non-traumatic groups were small and not significantly different, except for a small difference in thickness at total lung capacity (0.4 versus 0.3 respectively; P = 0.05). The association between the PFTs and diaphragmatic ultrasound measurements was unchanged when participants with non-traumatic injuries were excluded from the analysis.

## Discussion

This pilot study demonstrated the potential of diaphragmatic ultrasound as part of pulmonary surveillance for individuals with SCI. Current standards of care for monitoring pulmonary function include PFTs and fluoroscopic evaluation (6,9). Unfortunately, the time (37) required for these procedures and accessibility (11) to these tests are limiting factors for those with SCI. Diaphragmatic ultrasound presents a quick and accessible method for those managing individuals with SCI to monitor pulmonary function as a screening before determining if there is need for additional study. Previous work has demonstrated that 50–60% of vital capacity, in healthy individuals, is determined by the diaphragm (38) though this proportion varies in SCI depending on NLI (39). This provides a rationale for why thickening ratio, a direct measure of diaphragmatic function, had a moderate correlation with FVC (*r* = 0.5384). This is consistent with previous work that only evaluated those with NLI of C4-5 (22) and is clinically relevant because FVC has demonstrated diagnostic and prognostic value in chronic SCI (40) and is correlated well with many of the PFTs in those with tetraplegia (12). Previous work has demonstrated the diaphragm excursions during cough is correlated with PCF in healthy and intubated people (41,42). It is therefore reassuring that this correlation is consistent in individuals with SCI. Though PCF is more often associated with abdominal muscle strength, it is hypothesized that diaphragm excursion is correlated with PCF because inspiratory volume also determines cough strength (41). Diaphragm excursion is correlated with inspiratory volume (41) which explains why diaphragm excursion is also correlated with FVC. These correlations also align with previous work on PCF, which was utilized as a predictive marker for liberation from mechanical ventilation and tracheostomy decanulation (8) and chronically as a measure of cough strength (13,14). The ability to inhale (oxygenate), exhale (ventilation), and cough (to clear secretions) are important in staying off mechanical ventilation (43) and provides rationale for continued surveillance.

Findings from prior studies on the association between FVC and NLI are mixed. Ledsome and colleagues’ (44) study of adults with chronic, complete injuries finds that FVC is correlated with NLI (*n* = 16), whereas Roth and colleagues’ study of individuals injured fewer than 6 months (*n* = 52) did not (26). Our study did not find significant associations between NLI and the PFT or diaphragm ultrasound measurement outcome measures. Given the value of motor level in understanding function (25) we also assessed the association of the PFTs and diaphragmatic ultrasound measurements with motor level and did not find a significant association. Considering previous research has found that FVC (40,45) and PCF (46) are correlated with motor level, and that this study observed a correlation between PFTs and diaphragmatic ultrasound measurements, it was surprising an association between diaphragmatic ultrasound measurements and motor level did not reach significance. However, prior approaches assessed variation by subgroups of motor level (40), and the small sample size of this study precluded a full assessment of these differences as they fell within just one of these subgroups (40). Furthermore, the presence of asymmetry in diaphragmatic ultrasound measurements could also contribute to the challenge of detecting a significant correlation.

### Limitations

Although typical of a pilot study, the small sample size increases the risk of type 2 error which may contribute to some of the non-significant findings. Furthermore, the sample size increases the risk of selection bias and limits the generalizability of the data. Given this is a pilot and that levels C4-5 were more represented than others, this is expected and something to consider in future investigations.

Additionally, the normative data used in this research was based on healthy controls in a supine position (19,21). This study was performed with all patients reclined to 45° as has been performed in other research (18) because of the challenge of evaluating patients with neuromuscular disorders completely supine due to the impact of abdominal contents on diaphragm movement (43). However, normative values were not important because correlations were the priority, so the consistency rather than actual position was paramount. Additionally, the positions were not identical and respiratory capacities in individuals (especially those with neurological conditions) (9) varies by position which may have further impacted the associations.

Finally, PCF meters have been found to be less accurate than pneumotachograph for PCF < 270 L/min and may overestimate lower flow (47). Although a majority (9/14) of the subjects in this study had PCF <270 L/min, devices have improved since this was reported in 2004, and the device used in this study is the same as those in the hospital setting.

### Future direction

Given the significant correlations identified in this pilot study, a larger study would be beneficial to confirm the findings and to determine if there are significant correlations that may have clinically meaningful value (including a multivariate regression). For example, it is known that effort impacts PFTs (48) and, to a lesser extent, diaphragmatic ultrasound measurements (particularly thickening ratio) (19,49). It would be ideal to identify a diaphragm ultrasound measurement unimpacted by effort to correlate with a PFT, like thickness at functional residual capacity, to potentially remove effort as a variable. Although previous studies found it to be difficult to assess bilateral DE (32), it may be worthwhile to attempt because having both sides may provide a more representative picture of the asymmetry present in tetraplegia.

In addition, a larger study with more levels represented would be beneficial to determine if the normative values being used, based on healthy controls, are applicable to those with SCI. This is particularly important given the heterogeneity of SCI and how each level presents differently. It may be useful to include lower levels of injury including paraplegia to determine if the abdominal innervation present in these patients impacts the diaphragm excursion. Though no correlation with NLI or motor level were detected in this pilot, a larger study may detect a significant difference.

## Conclusion

This pilot study investigating the potential utility of diaphragmatic ultrasound as a tool of evaluating pulmonary function in individuals with cervical SCI demonstrated promise for this modality. Significant correlations were detected between thickening ratio with FVC as well as thickening ratio and diaphragm excursion with PCF. It would therefore be beneficial to expand this into a larger, longitudinal study to confirm these correlations and to assess whether normative values for NLI, motor level, or time since injury can be established so that providers can be equipped in interpreting and applying the results for individuals with SCI.

## Institutional review board (IRB)

The Kessler Foundation IRB approved this protocol. IRB Protocol Number: E-1240-23.

## Supplementary Material

Supplementary File List.docx

Supplemental material Diaphragm.docx
