# Supplementary material for: Correlation of diaphragmatic ultrasound with pulmonary function testing in patients with chronic cervical spinal cord injury: A single center pilot study
Source: J Spinal Cord Med. 2025 Jul 31;49(3):602–10. doi: 10.1080/10790268.2025.2534262 (PMC13123080; doi:10.1080/10790268.2025.2534262)
Supplement: Supplementary File List.docx [file YSCM_A_2534262_SM7801.docx]

Supplementary File List

| **File** | **Description** |
| --- | --- |
| e-Table 1 | Spearman rank correlation between NLI/Motor Level and PFTs and with diaphragmatic ultrasound measurements |

:
