# Supplementary material for: Correlation of diaphragmatic ultrasound with pulmonary function testing in patients with chronic cervical spinal cord injury: A single center pilot study
Source: J Spinal Cord Med. 2025 Jul 31;49(3):602–10. doi: 10.1080/10790268.2025.2534262 (PMC13123080; doi:10.1080/10790268.2025.2534262)
Supplement: Supplemental material Diaphragm.docx [file YSCM_A_2534262_SM7800.docx]

e-Table 1: Spearman rank correlation between NLI/Motor Level and PFTs and with diaphragmatic ultrasound measurements

| **Measurement (PFTs & Ultrasound Measurements)** |  | |
| --- | --- | --- |
| ***NLI*** | | |
| Forced Vital Capacity | Correlation coefficient (r) | 0.1355 |
|  | p- value | 0.6559 |
|  | | |
| Peak cough flow | Correlation coefficient (r) | 0.2521 |
|  | p- value | 0.4023 |
|  | | |
| Thickness at FRC | Correlation coefficient (r) | 0.3543 |
|  | p- value | 0.2326 |
|  | | |
| Thickness at TLC | Correlation coefficient (r) | 0.4336 |
|  | p- value | 0.138 |
|  | | |
| Thickening ratio** | Correlation coefficient (r) | 0.53 |
|  | p- value | 0.0633 |
|  | | |
| Diaphragm Excursion* | Correlation coefficient (r) | 0.3312 |
|  | p- value | 0.2662 |
| ***Motor Level***** | | |
| Forced Vital Capacity | Correlation coefficient (r) | 0.2377 |
|  | p- value | 0.4294 |
|  | | |
| Peak cough flow | Correlation coefficient (r) | 0.3144 |
|  | p- value | 0.2915 |
|  |  |  |
| Thickness at FRC | Correlation coefficient (r) | 0.1635 |
|  | p- value | 0.5891 |
|  | | |
| Thickness at TLC | Correlation coefficient (r) | 0.2033 |
|  | p- value | 0.5004 |
|  | | |
| Thickening ratio** | Correlation coefficient (r) | 0.295 |
|  | p- value | 0.3236 |
|  | | |
| Diaphragm Excursion*** | Correlation coefficient (r) | 0.0859 |
|  | p- value | 0.7774 |

*When the right and level motor levels were asymmetric, the lower of the two was used.

**Thickening ratio used for correlation when motor level was symmetric was the averaged thicknesses at TLC and FRC.

***Sample size for excursions is 13.
